# Advances in the measurement of vitamin D binding protein

**DOI:** 10.1007/s00216-026-06815-5

**Published:** 2026-09-15

**Authors:** Karen W. Phinney, Lisa E. Kilpatrick

**Affiliations:** 1 Biomolecular Measurement Division, Material Measurement Laboratory, National Institute of Standards and Technology, Gaithersburg, MD, USA

**Keywords:** Immunoassays/ELISA, Mass spectrometry, Metabolite, Protein quantification, Vitamin D, Reference materials

## Abstract

Vitamin D binding protein (DBP) is a polymorphic protein known for its function in transporting vitamin D metabolites. DBP also plays several other important roles in the body’s response to tissue damage and inflammation. Measurement of DBP is fundamental to understanding its relationship to vitamin D status and to elucidating potential links between DBP concentration and various health outcomes. Early studies on DBP primarily used antibody-based techniques for quantification, and mass spectrometry-based methods have been reported during the past decade. This review will outline the different methodologies used in DBP studies and to assign values to reference materials, describe their advantages and limitations, and highlight some future trends.

## Introduction

Vitamin D binding protein (DBP), originally known as group-specific component (Gc), is the principal plasma transport protein for vitamin D and its metabolites [1–3]. Vitamin D is essential for bone health, and deficiencies can lead to bone-related diseases in both children and adults [4, 5]. Vitamin D (either D_2_ or D_3_) undergoes hydroxylation in the liver to the corresponding metabolites, 25-hydroxyvitamin D_2_ [25(OH)D_2_] and 25-hydroxyvitamin D_3_ [25(OH)D_3_], which may then undergo further hydroxylation reactions [6]. DBP is produced in the liver and has a high binding affinity for vitamin D metabolites. Most of the circulating 25(OH) D (85% to 90%) is bound to DBP, with the remaining fraction (12% to 15%) loosely bound to albumin [7]. The term 25(OH)D is commonly used to refer to the sum of 25(OH) D_2_ and 25(OH)D_3_ concentrations. DBP is among the most abundant plasma proteins, and its concentration typically far exceeds the concentration of its vitamin D metabolite ligands. Most publications have indicated concentrations of DBP between 200 mg/L and 500 mg/L in human serum [1, 8, 9]. Pregnancy is associated with increased DBP concentrations [10], while liver and kidney diseases can result in decreased DBP levels [11]. DBP has several other biological functions apart from its role in transporting vitamin D metabolites that remain topics of investigation [12–15].

Human DBP is approximately 58 kDa in size, 458 amino acids long, and has significant sequence homology with albumin and α-fetoprotein [16, 17]. One of the most important early discoveries about DBP was its high degree of polymorphism [18, 19]. Although more than 100 variants of DBP have been identified [20], the three most common isoforms (Gc1f, Gc1s, and Gc2) arise from combinations of two single nucleotide polymorphisms (SNPs), rs7041 and rs4588, as shown in Fig. 1. The amino acid sequence variations also yield differences in glycosylation patterns among the three major variants [21, 22], which can be observed through changes in their electrophoretic mobility [23]. Attempts to quantify potential differences in binding affinities for vitamin D metabolites among the genetic variants of DBP have yielded conflicting results [24, 25].

Although the existence of DBP has been known since the late 1950 s, publications focused on elucidating the role of DBP in vitamin D status and overall health have increased significantly over the past two decades. Measurement of DBP has been an important element of many of those studies. This review will cover the various measurement methods that have been reported, describe their advantages and limitations, and outline potential future directions for DBP measurements. Some of the observations for DBP are also likely to be applicable to other proteins of clinical and nutritional interest.

## Why measure DBP?

Vitamin D status is commonly evaluated through the measurement of total 25(OH)D in either serum or plasma [26, 27]. Measurement of 25(OH)D generally involves a step where the metabolites are released from DBP, either through extraction or by displacement [28, 29]. Several studies have suggested that incomplete release of 25(OH)D from DBP in immunoassays may cause an underestimation of 25(OH)D concentrations, especially in individuals with higher DBP concentrations [30]. Muller Kobold et al. reported that elevated DBP concentrations in pregnant women were associated with a negative bias in 25(OH)D results from one commercial immunoassay when compared to results from a standardized liquid chromatography-tandem mass spectrometry (LC-MS/MS) method, and the bias was largely corrected when samples were pretreated with pepsin to ensure complete release of 25(OH)D [31]. Heijboer et al. compared results from several automated 25(OH)D immunoassays to an isotope dilution LC-MS/MS method and noted inaccuracies in some of the automated methods that seemed correlated to the DBP concentration [30]. A recent commutability study of NIST Standard Reference Materials^®^ (SRMs) [32] noted a lack of commutability or inconclusive commutability results for one or more levels of SRM 1949 Frozen Human Prenatal Serum. This four-level reference material, prepared from serum pooled from non-pregnant women and from women in each trimester of pregnancy, has assigned values for DBP concentration [10, 33]. Although the study was not focused on assay performance, the lack of commutability observed for most ligand binding assays at higher DBP concentrations in SRM 1949 suggests that measurement of DBP may be important in understanding the performance of 25(OH)D assays in certain populations.

Measurement of 25(OH)D continues to be the most widely used tool for assessing vitamin D status, although further hydroxylation of this metabolite in the kidneys is needed to produce the biologically active metabolite 1,25(OH)_2_D [6]. There has been a search for additional biomarkers that might shed light on possible connections between vitamin D exposure and conditions such as cancer, cardiovascular disease, and diabetes. Because the biological activity of steroid hormones, like vitamin D, is thought to be linked to the unbound form and not the total amount of hormone available (the free hormone hypothesis), a number of researchers have proposed that “free” or “bioavailable” 25(OH)D concentration could be a better indicator of vitamin D status than total 25(OH)D [34, 35]. Only a small percentage (< 1%) of 25(OH)D exists in a “free” state, not bound to any protein, while the term “bioavailable” 25(OH) D has been used to refer to any 25(OH)D not bound to DBP [3]. Direct measurement of free 25(OH)D is difficult because of its very low concentration (pg/mL) and the need for diffusion and dialysis steps [36]. A two-step immunoassay for free 25(OH)D is now commercially available and has been used to evaluate free 25(OH)D concentrations in several NIST SRMs [37]. An alternative approach is to calculate bioavailable 25(OH)D using equations incorporating the concentration of DBP, albumin, and affinity constants for the binding of 25(OH)D [38]. Hence, accurate measurement of DBP concentrations may be important for studies examining correlations between free or bioavailable 25(OH)D concentrations and various health outcomes [3, 39].

Measurement of DBP has also been used to investigate cases where individuals have severe vitamin D deficiency that does not respond to supplementation [40–42]. Henderson et al. reported undetectable levels of 25(OH)D in a patient with ankylosing spondylitis. The patient had failed to respond to any form of vitamin D supplementation, and levels of DBP were also below the limit of detection (< 3 μg/mL) of their mass spectrometry method. Genetic analysis revealed a homozygous deletion of the *GC* gene, resulting in an absence of DBP [40]. Banerjee [41] described a patient with very low 25(OH)D concentrations despite intensive supplementation. DBP was undetectable (< 10 μg/mL) by mass spectrometry, and further investigations revealed a loss-of-function variant that caused the absence of DBP. In these cases, measurement of DBP helped elucidate an underlying cause for apparent treatment-resistant deficiencies.

In addition to its well-established role in transporting vitamin D metabolites, DBP also plays an important part in managing the body’s response to tissue damage [43, 44]. DBP binds actin monomers released after tissue injuries and prevents their polymerization into filaments that can cause organ dysfunction. This process depletes the available supply of DBP, and decreased concentrations of DBP have been proposed as an indicator of severe trauma and sepsis [45–47]. Studies have also suggested potential links between DBP concentration and diabetes [48], neurological disorders [49], and multiple sclerosis [50], providing further evidence for the importance of DBP in understanding a variety of medical conditions.

## Antibody-based methods for DBP

Several groups reported measurement of DBP during the 1970 s as part of studies investigating the relationship between DBP concentrations and various health conditions. The methodologies generally employed some form of antibody-antigen binding to achieve quantification. Bouillon et al. used radial immunodiffusion to measure DBP in human serum and noted that DBP concentrations increased in women during pregnancy [51]. An antiserum against DBP was obtained from rabbits, and the diameter of ring-shaped precipitates formed by antibody-antigen interactions was used to quantify DBP. Haddad and Walgate developed a radioimmunoassay for DBP and did not observe any significant differences in DBP concentrations between vitamin D-deficient and vitamin D-supplemented individuals [52]. In their approach, displacement of ^125^I-DBP by human serum DBP could be used to detect DBP in very small (< 10 nL) serum sample volumes. Imawari and Goodman similarly developed both radial immunodiffusion and radioimmunoassays for measurement of DBP in serum [53]. They noted that patients with cirrhosis of the liver had reduced concentrations of DBP compared to normal subjects.

In the 1980 s, Walsh and Haddad leveraged the migration of DBP in an electric field to develop a “rocket” electrophoresis method for quantification [54]. Antiserum against DBP was included in the agarose gel, and the height of the rocket-shaped patterns of precipitation could be correlated with DBP concentration in serum. A decade later, an automated immunonephelometric assay for DBP was reported by Haughton and Mason [11]. In their approach, light scattering caused by the antibody-antigen complex was correlated in a linear manner with DBP concentration. They highlighted an improvement in precision for the automated methodology when compared to radioimmunoassay or radial immunodiffusion techniques. In 2004, Jorgensen et al. [55] reported an inhibition ELISA assay for measurement of DBP in serum and plasma. The method incorporated a mouse monoclonal antibody, in contrast to previous approaches that used polyclonal antibodies. They noted similar DBP concentrations in serum and plasma from the same individual and that DBP levels did not appear to be affected by freeze-thaw cycles. In their study, men had slightly lower DBP concentrations on average than women, and DBP concentrations increased in women during pregnancy.

## Potential limitations of antibody-based methods

As noted previously, concentrations of DBP can be used to estimate bioavailable 25(OH)D levels, and commercial immunoassays for DBP are now available. The polymorphic nature of DBP has sometimes been overlooked when employing these assays. A study by Powe et al. reported that Black Americans had lower circulating concentrations of both DBP and 25(OH)D on average than White Americans [56]. This observation led the authors to conclude that the two groups studied had equivalent levels of bioavailable 25(OH)D, despite the higher 25(OH) D concentrations generally found in White Americans. However, further scrutiny of the results suggested that the monoclonal antibody used in the DBP assay may not have equivalent binding to all three major phenotypes (Gc1f, Gc1s, Gc2), which could have caused a measurement bias. Subsequent studies using polyclonal antibodies have not found any significant differences in DBP concentrations between Blacks and Whites [57].

Antibody-based DBP assays are also subject to potential interference from actin binding, which causes a conformational change in DBP that could block certain epitopes recognized by antibodies [58, 59]. Hong et al. developed an immunoassay for DBP using antibody pairs that were unaffected by the presence of excess actin [45]. They confirmed that sepsis patients had lower DBP concentrations on average than healthy individuals.

## Mass spectrometry-based methods for DBP

Protein quantification through the use of “bottom-up” approaches is a well-established technique in mass spectrometric methods. The target protein is digested into its constituent peptides through the use of trypsin or another enzyme, and one or more peptides are quantified as surrogate indicators of the protein concentration [60, 61]. Henderson and coworkers developed an LC-MS/MS method for the measurement of DBP and compared their results to a commonly used monoclonal immunoassay [62]. Because DBP is one of the more abundant serum proteins, the LC-MS/MS method did not require affinity enrichment techniques. The authors employed two peptides (VLEPTLK and ELP-EHTVK) shared by all three common DBP proteoforms for quantification and a third peptide, THLPEVFLSK, for quality control. Use of the three peptides was intended to ensure that the results would not be biased by the presence or absence of a specific DBP isoform. They reported that DBP concentrations were similar in Black and White individuals when measured by the LC-MS/MS method; however, the monoclonal immunoassay results suggested significant differences in DBP concentrations between these two groups, similar to observations by other researchers [56]. The authors further suggested that the polymorphic nature of DBP and potential glycosylation sites might affect the performance of immunoassays targeting regions near the polymorphisms [9].

A similar LC-MS/MS method was described by Kilpatrick and Phinney [63] and used to examine DBP in a pooled plasma reference material (NIST SRM 1950 Metabolites in Frozen Human Plasma) that contained all three major DBP isoforms. Six peptides shared among the major DBP proteoforms were evaluated for quantification, and three were selected based on analytical reproducibility (see Table 1). Isotopically labeled peptides served as internal standards for quantification. The methodology was further applied to the assignment of a value for DBP concentration in SRM 1950 and in SRM 1949 Frozen Human Prenatal Serum [10, 64], making them the first reference materials available with assigned values for DBP.

Measurement of DBP has also been performed as part of efforts to estimate bioavailable 25(OH)D. Ko and coworkers reported a multiplexed LC-MS/MS method for the simultaneous quantification of DBP, albumin, 25(OH)D, and 24,25(OH)_2_D_3_ in serum [65]. In their method, a solution of guinea pig serum was used as an internal standard for DBP and albumin, and three peptides (LPDATPK, LPDATPTELAK, and LPEATPTELAK) were used to identify the DBP isoform present (see Table 1). An additional peptide, VLEPTLK, common to all three proteoforms, was used for DBP quantification.

One advantage of mass spectrometry methods that may not be immediately apparent is their ability to identify the haplotype of the individual without the need for genetic testing, if proteoform-specific peptides are included in the method. Henderson and coworkers compared the haplotype (Gc1s, Gc1f, and Gc2) identified by mass spectrometry with genotypic data from participants in the Atherosclerosis Risk in Communities (ARIC) study and noted > 98% accuracy in haplotype for the LC-MS/MS method [62]. Knowing both DBP concentration and phenotype might be beneficial for exploring indicators of vitamin D status beyond 25(OH)D concentrations [66].

## Potential limitations of mass spectrometry-based methods

Although mass spectrometry methods for DBP are not subject to the same potential sources of bias as antibody-based methods, they can be subject to other sources of experimental error, especially when the specific proteoforms present are not known in advance. Variability in digestion kinetics among the proteoforms could lead to different liberation rates of the target peptides and a bias in quantification. Kilpatrick et al. prepared external calibrants by spiking pooled human serum with DBP from homozygous donors and achieved similar quantitative results, regardless of which proteoform (Gc1s, Gc1f, and Gc2) was spiked into the pooled serum. These results, along with data comparing LC-MS/MS measurements of the same sample in two laboratories using different methods and instrumentation (Table 2), indicate that reproducible quantification of DBP is possible through careful peptide selection and method optimization [64].

Quantification of individual DBP proteoforms remains a challenge because of potential sites of glycosylation on the isoform-specific peptides. Isotopically labeled glycopeptides are not routinely synthesized for quantification, and both glycosylated and non-glycosylated forms of a given peptide may be found in the same sample [63, 67]. Glycosylated peptides often ionize less efficiently than their non-glycosylated counterparts [68], presenting a risk for measurement bias in LC-MS/MS quantification.

## Future trends

Measurement of DBP will continue to be an important part of understanding the complex role of vitamin D in health and disease. Studies have suggested a potential link between DBP concentration and the risk of certain autoimmune diseases, and further research may lead to new strategies for optimizing vitamin D status [69]. Immunoassays for DBP that are unaffected by the Gc phenotype present are likely to be the preferred choice for future studies when higher-throughput measurements are needed. However, there is a growing focus on potential associations between DBP phenotype and disease risk or outcomes. For example, a recent examination of reproductive outcomes suggested that one DBP variant might be associated with increased odds of live birth [70]. Potential associations between certain DBP variants and cancer risk were also highlighted by Rozmus et al. in a review article [71]. Future research in these areas may require combining an immunoassay with genotyping information or using an LC-MS/MS approach that identifies the proteoforms present to capture both DBP concentration and phenotype.

## Figures and Tables

**Fig. 1:**
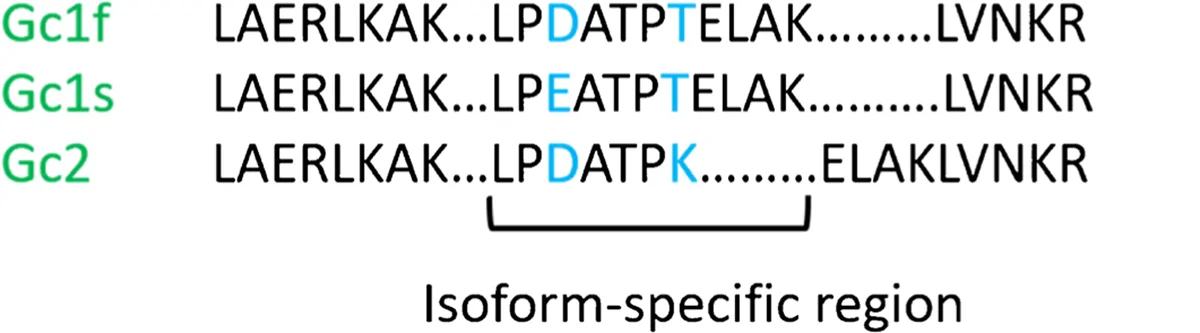
Partial amino acid sequences for the three most common variants of DBP. Specific variations in amino acid sequence are highlighted in blue. Adapted from reference [63]

**Table 1:** Tryptic peptides used for DBP identification and quantification

Peptide	Purpose	References

VLEPTLK	Quantify DBP	10, 39, 62, 63, 64, 65, 66, 70
ELPEHTVK	Quantify DBP	39, 62, 63, 66, 70
TSALSAK	Quantify DBP	10, 63, 64
LPEATPTELAK	Identify Gc1s	62, 63, 65, 66, 70
LPDATPTELAK	Identify Gc1f	62, 63, 65, 66, 70
LPDATPK	Identify Gc2	62, 63, 65, 66, 70

**Table 2:** Comparison of DBP concentration results from NIST and the University of Washington

Vial No.	DBP concentration (μmol/kg) in SRM 1950 at NIST^a^	DBP concentration (μmol/kg) in SRM 1950 at UW^a^

1	3.13 (0.73)	3.58 (3.23)
2	3.19 (2.59)	3.51 (2.40)
3	3.13 (1.95)	3.49 (2.98)
4	3.17 (3.63)	3.44 (3.09)
5	3.25 (5.64)	3.49 (2.24)
6	3.12 (3.26)	3.50 (2.53)
7	3.17 (3.81)	3.47 (1.38)
Mean	3.17 (3.44)	3.50 (2.68)

a Two sample aliquots from each vial were prepared and analyzed on three different days. Mean values (*n* = 6) for each vial are shown (%CV in parentheses). The overall mean value for each lab (NIST or UW) represents *n* = 42. Adapted from reference [64]
